# Optimization of *Oliveria decumbens* essential oil nanoemulsion formulation using RSM and development of a bioactive film based on *Plantago ovata* mucilage

**DOI:** 10.1039/d5ra06562c

**Published:** 2026-01-28

**Authors:** Azita Shafiei, Javad Safaei-Ghomi, Mehdi Mehran

**Affiliations:** a Department of Organic Chemistry, Faculty of Chemistry, University of Kashan Kashan 87317-51167 I. R. Iran safaei@kashanu.ac.ir +98-31-55552935 +98-31-55912385; b Barij Medicinal Plants Research Centre Kashan I. R. Iran

## Abstract

The aim of this study was to optimize the conditions for the preparation of *Oliveria decumbens* essential oil (OEO) nanoemulsion by response surface methodology using ultrasonic technique. Considering droplet size as the response, the optimal conditions for nanoemulsion fabrication were obtained with a sonication amplitude of 21.2%, sonication time of 9.53 min and surfactant-to-oil ratio of 3.88. Under these conditions, the particle size of the nanoemulsion was 76.6 ± 0.81 nm, the polydispersity index was 0.206 ± 0.02, and the zeta potential was −1.45 ± 2.15 mV. The concentrations of oil and surfactant were found to significantly influence the droplet size of the nanoemulsion. The optimized nanoemulsion was characterized for its antioxidant, antimicrobial activity and morphology. In addition, a bioactive edible film based on *Plantago ovata* gum incorporating 5, 10 and 15% OEO nanoemulsion was developed. Mechanical and physical characteristics of prepared films namely film thickness, tensile strength, water solubility, water vapor permeability were evaluated. The elongation at break increased and the tensile strength of the films significantly decreased when the nanoemulsion was incorporated. Considering the antimicrobial and antioxidant properties of the fabricated edible films based on *Plantago ovata* gum and OEO (POMG/OEO), they have good potential for extending the shelf life of food products.

## Abstract 2

The aim of this study is to optimize the conditions for the preparation of *Oliveria decumbens* essential oil (OEO) nanoemulsion by response surface methodology using ultrasonic technique.

## Graphical abstract

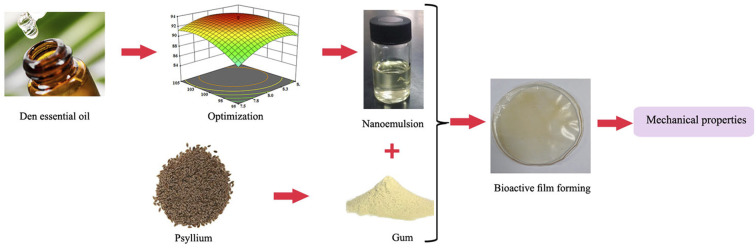

## 1 Introduction

*Oliveria decumbens* Vent, with the local common names of “Denak”, “Den”, and “Mooshkorok” is one of the native plants of Iran that grows in the southern and south western region of the country.^1^ Traditionally, the aerial parts of this plant are used for the treatment of abdominal pain, indigestion, diarrhea, and fever.^2^*Oliveria decumbens* essential oil (OEO) contain several bioactive substances including thymol, carvacrol, γ-terpinene, and *p*-cymene.^3^ The amount of these monoterpene compounds varies depending on the climatic conditions, geographic origin, and harvest season.^4^ The OEO also possesses anticancer,^5^ anti-inflammatory,^6^ antimicrobial,^7^ and antioxidant^8^ properties.

The use of artificial antioxidants and antimicrobials in the food industry increases the risk of toxicity and cancer.^9^ Therefore, today, the interest in using natural antimicrobial agents such as essential oils (EOs) has increased.^10^ In addition, EOs have shown potential anti-inflammatory,^11^ antifungal,^12^ antioxidant,^13^ anti-cancer^14^ and anti-tumoral activities.^15^ However, the use of EOs faced various challenges such as sensitivity to light and temperature, and their strong odor/flavor. In addition, their low solubility in water and potential toxicity at high doses are its main limitation.^16^ These challenges and limitations can be greatly reduced by using nanoemulsion systems. In fact, by reducing the size to the nanoscale, biological activities are improved due to an increased surface-to-volume ratio. Furthermore, it becomes easier to pass through the biological membranes, which leads to an increase in their bioavailability.^17^ Moreover, studies have shown that converting EOs into nano form significantly improves their properties of the EOs such as physical stability, appearance, and antimicrobial activity.^18,19^ Low energy and high energy emulsification methods are two approaches for nanoemulsion preparation. The high energy method uses mechanical forces reduce particle size, while the low energy method uses the energy stored in the system to produce nanosized droplets.^20^ Microfluidizers, ultrasonicators, and high-pressure homogenizers are devices that can produce nanoparticles in high energy approach.^21^ Low-energy methods include phase inversion and spontaneous emulsification.^22^

In past decades, the common approach to response optimization was the one-variable-at-time method, in which one variable was investigated while the others remained constant. The disadvantages of this method include requiring a large number of experiments, ignoring factor interactions, and being time-consuming. Response surface methodology (RSM) is one of the multivariate techniques that describes relationships between independent variables and responses using statistical modeling. Central Composite Design (CCD) is the most widely used response surface design for formulation optimization.^23–25^

Psyllium (*Plantago ovata*) is well recognized for its pharmacological effects such as wound healing, anti-constipation, anti-diarrheal, hypocholestrolemic and hypoglycemic activities. The outer seed coat of psyllium contains 10–30% of hydrocolloid which can be separated into acidic and neutral polysaccharides.^26^*Plantago ovata* mucilage has various pharmaceutical applications due to its disintegrating, binding, film forming, emulsifying, suspending and thickening properties and it is particularly attractive because of its low cost and plant origin. This mucilage due to being a good source of soluble and insoluble fiber, and possessing antioxidant and antibacterial properties, could be a potential candidate for edible film production.^27,28^

Recently, the use of biopolymer films packaging materials as an alternative to conventional synthetic polymeric packaging, has attracted considerable attention due to the destructive effects of plastics on the environment. Bioactive films are composed of various edible components such as proteins, lipids, polysaccharides, or their combinations. Natural polymer-based films offer several advantages including being as environmentally friendly, biocompatible and affordable. However, they often exhibit low water resistance and poor mechanical properties. Incorporating essential oils due to their antimicrobial and antioxidant activities can be a promising approach for the preparation of bio-based films.^29^

To the best of our knowledge, the incorporation of OEO nanoemulsion into psyllium mucilage-based films has not been reported yet. The purpose of the present work was to optimize the formulation of OEO nanoemulsion using RSM and ultrasonic technique. In the next stage, antimicrobial and antioxidant activities of the optimized nanoemulsion were evaluated. Finally, a new edible film based on psyllium mucilage was prepared using the optimized nanoemulsion, and evaluate its physicochemical properties.

## 2 Materials and methods

### 2.1 Materials

Pure OEO and *Plantago ovata* were donated from Barij Essence Pharmaceutical Company. PEG-40 hydrogenated castor oil CO 40 (HCO) was commercially available product from Mosselman Chemical Company (Ghlin, Belgium). 2,2-Diphenyl-1-picrylhydrazyl (DPPH), 2,4,6-tri(2-pyridyl)-*s*-triazine (TPTZ) were obtained from Sigma Chemical Co. (St. Louis, MO). Polyethylene glycol 400 (PEG 400) and Tween 80 (polyoxyethylene-20-sorbitan-monooleate) were purchased from Kimyagaran Emrooz Company.

### 2.2 Preparation of OEO nanoemulsions

All emulsions were made by high energy method and in two steps. OEO, HCO, Tween 80 were selected as the oil phase, and PEG 400 as cosolvent were added to water. After titration of the oily phase with the aqueous phase at a speed of 1000 rpm, the coarse emulsion was then subjected to ultrasonic emulsification with a 20 mm diameter tip horn for 5 minutes at room temperature (HD3200, 20 kHz, Bandelin, Berlin, Germany), and the ultrasonication was carried out under an ice bath. The prepared nanoemulsions were kept in amber glass at room temperature until further analysis.

### 2.3 Experimental design

A three-factor central composite design (CCD) was used to determine the effect of the value of surfactant-to-oil ratios (SORs) (1–4%, *X*_1_), time (5–12, *X*_2_), and applied power (30–55, *X*_3_), on droplet size of nanoemulsion as response variables. So, according to the central composite design (CCD), 20 experimental runs were generated by using Design-Expert 11.1.0 software (Table 1). For this design, a second-order polynomial equation was employed to explain droplet size (*y*) of the nanoemulsions as a function of the independent variables:

1 *y* = *β*_0_ + *β*_1_*X*_1_ + *β*_2_*X*_2_ + *β*_3_*X*_3_ + *β*_11_*X*_1_^2^ + *β*_22_*X*_2_^2^ + *β*_33_*X*_3_^2^ + *β*_12_*X*_1_*X*_2_ + *β*_13_*X*_1_*X*_3_ + *β*_23_*X*_2_*X*_3_

where *β*_0_ is a constant term; *y* is the predicted response; *β*_1_, *β*_2_ and *β*_3_ are the linear, quadratic and interaction coefficients, respectively. A multiple regression technique was used to analyze the experimental data. After eliminate insignificant values (*P* > 0.05), polynomial regression equation was generated and contour plots were plotted. With the help of this equation, the optimized conditions for the preparation of nanoemulsion were calculated.

**Table 1:** Droplet size of OEO nanoemulsions obtained from central composite design

Run	Independent variable			Droplet size (nm)	
	*X*_1_	*X*_2_	*X*_3_	Experimental	Predicted
1	2.5	8.5	42.5	124.3	123.9
2	4.0	5.0	30.0	109.7	107.4
3	4.0	5.0	55.0	147.5	150.1
4	1.0	5.0	30.0	147.6	147.2
5	2.5	8.5	21.5	112.6	111.6
6	1.0	5.0	55.0	180	182.2
7	0.0	8.5	42.5	175.2	176.3
8	2.5	8.5	42.5	116.1	115.6
9	2.5	2.6	42.5	153.8	153.1
10	4.0	12.0	55.0	97.3	98.4
11	5.0	8.5	42.5	76.8	76.2
12	2.5	14.4	42.5	101.2	101.3
13	2.5	8.5	42.5	126.7	127.6
14	2.5	8.5	42.5	126.3	125.3
15	2.5	8.5	63.5	166.8	165.7
16	2.5	8.5	42.5	119.3	120.4
17	4.0	12.0	30.0	84.4	85.4
18	2.5	8.5	42.5	117.9	118.3
19	1.0	12.0	30.0	139.1	140.2
20	1.0	12.0	55.0	163.6	162.1

### 2.4 Droplet size measurement

The electrophoretic features of prepared nanoemulsions consisted of droplet size, polydispersity index (PDI), and zeta potential, were carried out applying the dynamic light scattering (DLS) instrument (Malvern Instruments, UK). All measurements were performed at scattering angle 90° and 25 °C.

### 2.5 Antioxidant activity measurement

#### 2.5.1 DPPH radical scavenging activity

DPPH method is based on the reduction of methanolic DPPH solution in the presence of hydrogen donating antioxidant. The antioxidant activity of the pure OEO, nanoemulsion and bioactive film was determined using the diphenyl-2-picrylhydrazyl (DPPH) radical scavenging assay. For this purpose, 2 mL of extracted solution was added to 2 mL of DPPH with concentration 60 µL mL^−1^, and after keeping it in the dark for one hour, its absorbance was recorded at 517 nm. In this experiment, methanol and DPPH (60 µL mL^−1^) was employed as blank and control sample, respectively. The value of specimen leading to 50% inhibition of DPPH free radicals (IC_50_) was calculated considering to the absorbance at mentioned wavelength *versus* concentration of the sample. By using eqn (2), the radical scavenging activity (% SA) can be calculated:2
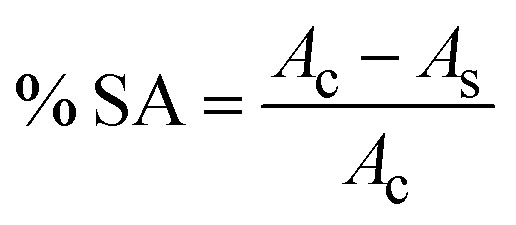
where *A*_s_ and *A*_c_ are the absorbance values of the sample solutions and the DPPH in methanol as control solution, respectively.

#### 2.5.2 Ferric ion reducing antioxidant power (FRAP) assay

Ferric reducing antioxidant potential (FRAP) assay was estimated using the FRAP assay Benzie *et al.* with a few modifications.^30^ This method is based on the reduction (Fe^3+^-TPTZ) complex to ferrous (Fe^2+^) form at low pH. FRAP reagent is a mixture of 50 mL of 300 mmol L^−1^ acetate buffer adjusted to pH 3.6, and 5 mL of 20 mmol L^−1^ ferric chloride, and 5 mL of 10 mmol L^−1^ TPTZ in 40 mmol L^−1^ hydrochloric acid. This reagent must be prepared freshly. 1 mL of each sample and 0.3 mL distillate water were allowed to react with 3 mL of the FRAP solution and the reaction was completed by putting into water bath (37 °C) for 30 min. Then, the absorbance of the reaction mixture was recorded at 593 nm by spectrophotometer (PerkinElmer Lambda EZ-210). A blank contains all reagents except the sample solution. All measurements were repeated three times. FRAP values were reported as µM Fe^2+^ per g of sample.

### 2.6 Transmission electron microscopy (TEM)

Philips CM300 TEM (Netherlands) apparatus was used to determination morphology and size of nanoemulsion. The nanoemulsion was placed in the specific cell and covered with a 200 mesh grids. After the sample was dried, it was observed under the transmission electron microscope and relevant images were recorded.

### 2.7 Gas chromatography analyses

Evaluation of the pure OEO and OEO nanoemulsion compounds were carried out using gas chromatography analyzer (CP-3800, Varian, Netherlands) with flame ionization detector. The analysis was performed with a CP-Sil 8 CB capillary column (0.32 mm internal diameter, 60 m length, and 0.25 µm film thickness). The EO sample was injected to GC system by split ratio 1 : 50. The temperature of the column was raised to 250 °C with a rate of 4 °C min^−1^ and was held at 250 °C for 15 min. The principal compounds of EO were recognized by comparing them with the retention times of standard compounds from Carl Roth Company in capillary column.

### 2.8 Determination of minimum inhibitory concentration (MIC) and minimum bactericidal concentration (MBC)

Minimum inhibitory concentrations (MICs) are evaluated the ‘gold standard’ for determining the susceptibility of microorganisms to antimicrobials. The MIC is defined as the lowest concentration of a drug that will inhibit the visible growth of an organism at specific time. In this study, we evaluated the antimicrobial activity of the OEO and its optimized nanoemulsions formed against five different microbial strains include *Staphylococcus aureus* (ATCC 29737), *Escherichia coli* (ATCC 25922), *Bacillus subtilis* (ATCC 6633), *Salmonella paratyphi*-A serotype (ATCC 5702), *Aspergillus niger* (ATCC 9029). Briefly, 100 µL of diluted microbial suspension (10^6^ CFU mL^−1^) were inoculated to each well, and 100 µL of diluted OEO in DMSO in media broth (1–5 µL mL^−1^) were added and incubated for 24 h at 37 °C. The first well that had no visual turbidity in media was defined as the MIC and the first well that had no growth on nutrient agar was determined as MBC.

### 2.9 Preparation of edible films *Plantago ovata* mucilage gum incorporated with OEO nanoemulsions (POMG/OEO)

The 50 g *Plantago ovata* seed was mixed with 1 L of water and filtered after 24 h. The obtained gel was mixed with 1.5 L of 96% ethanol until the mucilage precipitated completely. The precipitate was separated using a centrifuge at 5000 rpm. *Plantago ovata* mucilage gum (POMG) was washed at dried at 45 °C. For preparation of POMG film, appropriate amount of PMOG was added gradually into water already containing 5% glycerin and 10% gelatin, stirred and heated to 80 °C at 1200 rpm for 10 min. POMG dispersion was mixed with optimized OEO nanoemulsions to reach three different concentrations (5%, 10% and 15%, w/w) and homogenized for 15 minutes at 1500 rpm (Heidolph, Germany). After debubbling, the solution was poured into plexiglass plates (260 mm × 260 mm) and was placed in an oven 45 °C for approximately 30 h.

### 2.10 Characterization of POMG/OEO film

#### 2.10.1 Moisture content

Moisture content of films was calculated by gravimetric method, for this purpose, the film for drying was placed in an oven 90 °C to reach fixed weight, after weighing, the moisture content was calculated.

#### 2.10.2 Film thickness

Thickness measurements were made at ten random locations of the film's sheets. A digital micrometer (Mitutoyo, Japan) with an accuracy of 0.001 mm was used for this purpose.

#### 2.10.3 Determination of water vapor permeability (WVP)

WVP of films was done according to ASTM E96 standard method with small modification.^31^ A desiccator containing a saturated magnesium solution was prepared using magnesium nitrate salt. The films were cut into circles and sealed onto glass cups containing deionized water. The cups were placed in a desiccator and the weight loss was recorded over 24 hours. WVP was calculated as the following equation:3
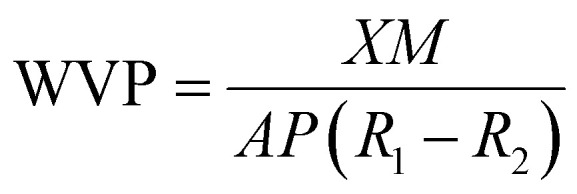
where *A* (m^2^) is the film area, *X* (m) is the film thickness, *M* is the curve slope, *P* (Pa) is the saturation vapor pressure of water, *R*_1_ and *R*_2_ are the RH in the cup and the desiccator, respectively.

#### 2.10.4 Mechanical properties

Before determining of mechanical film properties, all film strips were stored in a storage room temperature (25 °C) and humidity (53% RH) for 48 h to distribute the moisture evenly throughout the film and reach equilibrium. A texture analyzer (Brookfield CT3, USA) apparatus was used to evaluation mechanical properties such as tensile strength and elongation at break (*E*).

## 3 Results and discussion

### 3.1 Experimental design

The droplet size of the OEO nanoemulsions obtained from twenty the experiments are given in Table 1. Upon analysis of the measured values by analysis of variance (ANOVA), a quadratic model with high regression was fitted to data (*R*_adj_ = 0.9697). In order to evaluate of adequacy of model, normal probability plot of the residuals and plot of predicted droplet size *versus* actual their values were investigated. The predicted droplet size matched well with the experimental ones obtained from the quadratic model. Finally, the fitted quadratic polynomial equation for droplet size as a function of independent variables is:

4 *y* = 199.74 − 13.40*X*_1_ − 2.14*X*_2_ − 1.80*X*_3_ + 1.21*X*_12_ + 0.26*X*_22_ + 0.04*X*_32_ − 1.09*X*_1_*X*_2_ − 0.012*X*_1_*X*_3_ − 0.09*X*_2_*X*_3_

The independent variables that greatest impact the droplet size of the nanoemulsion were SOR, followed by the ultrasonic time. The negative value in the equation shows an effect the inverse relationship between the response and the factor, which means that as the three independent variables increase in the linear term, the amount of droplet size decreases. A higher SOR provides greater surfactant coverage at the oil–water interface, lowering interfacial tension and facilitating the formation of smaller droplets through enhanced steric stabilization and prevention of coalescence (Ostwald ripening). Similarly, prolonged ultrasonication time and higher power increase cavitation energy, generating intense shear forces and turbulent flow that disrupt larger droplets into nanosized ones.^24^

According to eqn (4) and investigating the interaction effect between independent variables, the production conditions for nanoemulsion production will be at surfactant oil ratio 3.88, time of 11.92 minutes and ultrasonic power of 32.86 watts. This RSM-based approach not only empirically optimized the formulation but also elucidated the interplay between formulation (SOR) and process parameters (time and power).

### 3.2 Analysis of variance

Table 2 shows the impact of independent variables on the droplet size of nanoemulsions. For any of the terms in the equation, a *p*-value smaller than 0.05 and large *F*-value indicates a significant effect of that variable on the relevant parameter. *P*-Values of regression and lack of fit suggest that the model has the suitable adequacy. Also, the terms *X*_1_*X*_1_, *X*_2_*X*_2_, *X*_1_*X*_3_, *X*_2_*X*_3_ do not have a significant effect on the size of nanoemulsion droplets. Therefore, they can be removed from the equation. This indicates that the effects of the independent variables on droplet size were predominantly linear. Also, the findings align with ultrasonic nanoemulsification mechanisms: higher SOR reduces interfacial tension and enhances steric stabilization, while longer time and greater power intensify cavitation and shear forces.^32^

**Table 2:** Analysis of variance for process optimization of OEO nanoemulsion formation

Source	DF	Adj SS	Adj MS	*F*-Value	*P*-Value
Regression	9	16 017.9	1779.8	64.96	0.0001
*X*_1_	1	8867.9	8867.8	323.65	0.0001
*X*_2_	1	2342.64	2342.64	85.50	0.0001
*X*_3_	1	2813.68	2813.68	102.61	0.0001
*X*_1_ *X*_1_	1	91.12	91.12	3.13	0.1015
*X*_2_ *X*_2_	1	118.91	118.91	4.34	0.0669
*X*_3_ *X*_3_	1	669.8	669.8	24.45	0.008
*X*_1_ *X*_2_	1	254.32	254.32	9.28	0.0139
*X*_1_ *X*_3_	1	0.441	0.441	0.016	0.9018
*X*_2_ *X*_3_	1	105.61	2813.68	3.85	0.0812
Lack of fit	5	150.86	30.17	1.26	0.4231
Pure error	4	95.73	30.17		
*R* ^2^	0.9848				
Adjusted *R*^2^	0.9697				
Predicted *R*^2^	0.8965				

### 3.3 Response surface analysis

**Fig. 1:**
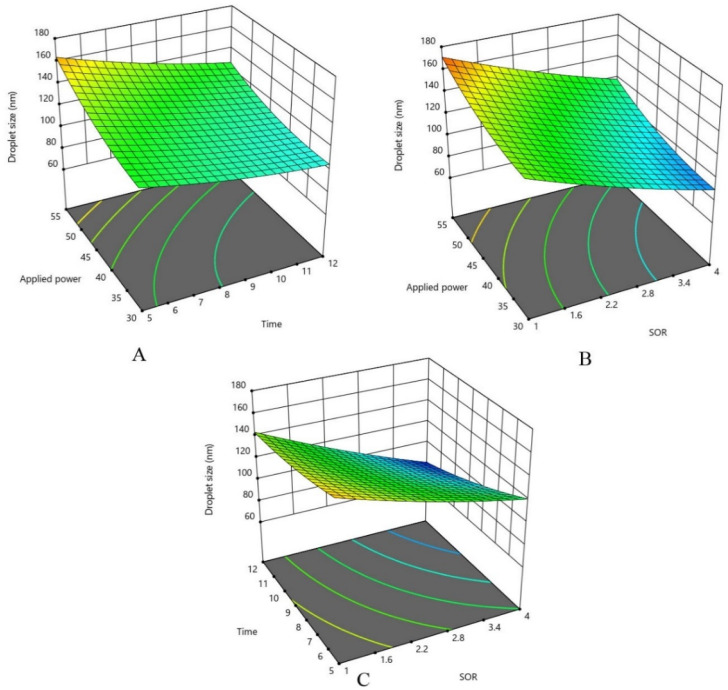
3D surface for droplet size of OEO nanoemulsion: (A) with middle value of SOR (B) with middle value of time, (C) with middle value of applied power.

In pharmaceutical and food industries, minimizing droplet size in nanoemulsions is crucial, as it enhances aqueous solubility, physical stability, controlled release of bioactives, and bioavailability while reducing potential side effects. To interpret the interaction effect of variables on droplet size, response surface was plotted by changing two variables while the third variable is kept constant at the central point. Fig. 1(B) demonstrate that the droplet size decreases with increasing SOR. The reason for this can be related to the complete coverage of the emulsifier on the new drops leads to a decrease in surface tension at the oil/water interface.^33^ Also, sufficient surfactant as a critical factor in emulsification processes, coverage provides steric or electrostatic repulsion, preventing droplet coalescence and stabilizing newly formed interfaces. In contrast, Fig. 1(A and C) demonstrate optimal values for ultrasonication time and power, beyond which droplet size increases. Moderate increases in power generate greater shear forces through intensified acoustic cavitation—collapsing microbubbles that produce localized turbulence and disruptive energy, effectively fragmenting larger droplets into nanosized ones. However, excessive ultrasonication time or amplitude can induce elevated local temperatures, accelerated molecular mobility, and recoalescence of droplets.

There is an optimal value for both time and applied power, which means that the size of the particles decreases and then increases with the increase of the amplitude. Increasing the *X*_3_ term (applied power) can create more shear force, which can reduce the droplets size of emulsion. On the other hand, a large increase in ultrasonic time and amplitude leads to an increase in the coalescence of emulsion droplets, which causes an increase in particle size. Similar result between applied power and droplet size was also observed for the preparation of eucalyptus oil nanoemulsion^34^ and designing curcumin loaded nanoemulsion,^35^ where over-processing led to recoalescence and larger droplets.

### 3.4 Mean droplet size

The average droplet size, PDI, and zeta potential of OEO are presented in Table 3. Optimized formula had a droplet size of 76.6 nm. The typical particle size range for nanoemulsions is between 10 and 500 nm, so the results obtained confirm that ultrasonic emulsification technique have been successfully applied in the nanoscale range. This parameter could be very important for physiochemical characterization which includes drug releasing and stability of nanoemulsions.^36,37^

**Table 3:** Characterization of optimized OEO nanoemulsion

Sample	Room temperature (25 °C)	Centrifugation (5000 rpm)	Size (nm) ± SE	PDI ± SE	Zeta potential (mV)
OEO nanoemulsion	Ok	Ok	76.6 ± 0.81	0.206 ± 0.02	−1.45

PDI represents the uniformity, homogeneity and stability of particles in the nanoemulsion. The PDI values of nanoemulsions prepared in this study was obtained 0.206. When this parameter is smaller than 0.3, it means that the nanoemulsion system has a narrow particle size distribution and is more stable.^24^ Another indicator used to evaluate physical stability of colloidal systems is zeta potential. Sufficiently negative values (above 25 mV) provide stability against factors such as such as coalescence and flocculation. In this study, due to the use of Tween 80 and HCO as emulsifier, both of which are non-ionic, no significant negative charge was generated.

### 3.5 Identification of OEO nanoemulsion chemical components

Analysis of chemical compounds of OEO through GC/FID showed the presence of 12 key compounds. The main compounds included thymol, *p*-cymene and γ-terpinene 26.1%, 13.8% and 11.3%, respectively. Other OEO compounds included limonene (5.1%), carvacrol (4.7%), myristicin (4.4%), β-phellandrene (2.1%), myristicin (2.8%), and terpinolene (0.5%). Alizadeh Behbahani *et al.* reported that thymol (28.45%) was the major component in the OEO. Other essential oil compounds included γ-terpinene (22.2%), *p*-cymene (17.90%), myristicin (13.55%), carvacrol (8.50%).^7^ Another study conducted by Hajimehdipoor *et al.* showed that the main ingredients of the OEO include γ-terpinene (23.33%), thymol (20.46%), myristicin (21.68%), *p*-cymene (19.40%) and carvacrol (9.45%).^1^ The composition of essential oils changes with the variation of some conditions such as harvest season, geographical diversity, storage conditions, *etc.* Therefore, the amounts of compounds in some studies are different, but in all reports, thymol is one of the main components.^38^ Thymol is the key component in the den and thyme essential oil. The compound is a natural phenolic monoterpene that has antitumor, antiviral, antibacterial, and anti-inflammatory properties.^39,40^ Most of the properties of OEO can be related to the presence of this important compound.^41^

### 3.6 TEM observation of droplets

TEM was used to observe the OEO droplets in the best formulated nanoemulsion. The TEM image of the nanoemulsion droplets showed with a smooth surface, almost uniform distribution and spherical shape. These morphological features are characteristic of a stable oil-in-water (O/W) nanoemulsion system, in which the surfactant layer efficiently encapsulates the oil droplets, thereby preventing coalescence and preserving long-term structural integrity. The sizes of droplet were congruent with those the results from droplet size analysis by DLS in the previous section. Overall, the favorable morphology confirmed by TEM underscores the physical stability of the formulated nanoemulsion and highlights its promising potential for applications in food preservation and pharmaceuticals.

### 3.7 Analyzing antioxidant activity

The DPPH radical (DPPH˙) can accept hydrogen atoms from antioxidants, forming a stable, non-radical DPPH-H molecule. This reduction results in the formation of the stable DPPH-H, which changes the color of the reaction solution from purple to yellow, thereby reflecting the radical scavenging capacity of the sample.

The radical scavenging activity of selected nanoemulsions was assessed. The antioxidant properties of mentioned samples estimated by DPPH and FRAP method are presented in Table 4. The concentration that resulted in 50% inhibition (IC_50_) was 52.7 µg mL^−1^ for the OEO and 60.9 µg mL^−1^ for the corresponding nanoemulsion. Considering the IC_50_ and comparing the antioxidant activities with butylated hydroxytoluene (IC_50_ = 21.5) as standard commercial synthetic antioxidant, we conclude that the OEO and nanoemulsion show good antioxidant activity.

**Table 4:** Antioxidant activity of OEO and its nanoemulsion and standard antioxidant using DPPH and FRAP method

Antioxidant	DPPH, IC_50_ (µg mL^−1^)	FRAP (µmol Fe^2+^ per g)
OEO	52.7	61 342
OEO nanoemulsion	60.9	60 458
BHT	21.5	46 250

Also, regarding that lower amounts have stronger antioxidant activity, this characterization is higher for free EO.

Antioxidants that scavenge radicals are often powerful reducing agents. Their capacity to transfer electrons to radicals is commonly assessed using FRAP assays. Reducing power is typically linked to the presence of reductants that exhibit antioxidant properties, as they interrupt the free radical chain by donating a hydrogen atom. Consequently, a strong general correlation is expected between reducing power and the DPPH radical scavenging activities. Thus, the OEO, which showed stronger antioxidant properties in DPPH assay, showed greater reducing power in FRAP assay. These findings were in tandem with other previous reports. As such, Sampaio *et al.* showed that the antioxidant properties of thyme essential oil are reduced after encapsulated into nanoemulsions by low-energy method.^9^ In contrast, some studies showed that the antioxidant activity of *Citrus medica* and *Cymbopogon densiflorus* essential oils was higher when they were converted into nanoemulsions.^42,43^

### 3.8 Antibacterial activity

The MIC and MBC values of a nanoemulsion in comparison to pure OEO, was reported in Table 5. The effect of the nanoemulsion system on the antimicrobial activity depended on the target microorganism. For *Bacillus subtilis* and *Aspergillus niger*, the MIC and MBC values were reduced from 1.32 and 1.8 µg mL^−1^ in OEO to 1.16 and 1.0 µg mL^−1^ in nanoemulsion, respectively. While the values were increase from 1.128 in OEO to 1.64 µg mL^−1^ in nanoemulsion for *Salmonella paratyphi*-A serotype. Interestingly, nanoemulsification of OEO did not significantly change the MIC and MBC values for *Staphylococcus aureus* and *Escherichia coli*.

**Table 5:** MIC and MBC measurements of OEO and its nanoemulsion on different microbial strains

	OEO		Nanoemulsion	
	MIC	MBC	MIC	MBC
*Staphylococcus aureus*	1.32	1.32	1.4	1.4
*Escherichia coli*	1.32	1.32	1.4	1.4
*Salmonella paratyphi*-A serotype	1.128	1.128	1.64	1.64
*Bacillus subtilis*	1.32	1.32	1.16	1.16
*Aspergillus niger*	1.8	1.8	1.0	1.0

These differential outcomes underscore that converting essential oils into nanoemulsions does not universally enhance antimicrobial activity but is modulated by multiple factors, including the nanoemulsion preparation method, the physicochemical characteristics of the EO, and the intrinsic properties of the target microorganisms. The different results are influenced by many factors such as method of preparation of nanoemulsion, type of test microorganism, and characterizations and compositions of EOs. For example, the enhanced antimicrobial activity against *B. subtilis* and *A. niger* can likely be attributed to the nanoemulsion's smaller droplet size, which significantly increases the surface area-to-volume ratio. This facilitates improved diffusion across microbial membranes and promotes more effective interactions with intracellular targets.^44^

Conversely, the diminished efficacy against *S. paratyphi*-A could be attributed to the outer lipopolysaccharide layer in Gram-negative bacteria, which might hinder the release or penetration of encapsulated EO, or to potential interactions between the surfactant for example Tween 80 and bacterial efflux systems that reduce intracellular accumulation. The lack of significant change in antimicrobial activity against *S. aureus* and *E. coli* is consistent with reports indicating that nanoemulsions can stabilize essential oils without necessarily enhancing their potency.^45^

These findings are consistent with the heterogeneous results reported in the literature. For example, Moghimi *et al.* have founded that nanoemulsion of *Thymus daenensis* EO shows 10 times more antibacterial activity than the its pure EO.^46^ While in Yazgan's research, it was revealed that sage essential oil is slightly more effective on microorganisms tested than its nanoemulsion form.^47^

### 3.9 Physical characteristics of edible films containing OEO

**Fig. 2:**
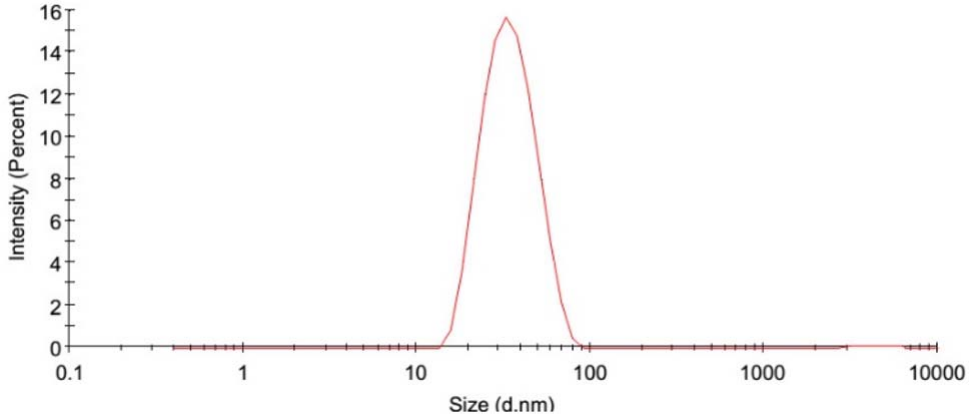
Particle size distribution of optimized OEO nanoemulsion.

Physical and mechanical properties of edible films are shown in Table 6. The control film had a moisture of 12.12 ± 0.47%, which decreased significantly with addition of OEO concentration. The reduced film moisture content could be due to hydrophobicity and volatility of EO compounds. Similar results were reported for moisture potato starch containing *Oregano* EO edible film^48^ and citral and quercetin incorporated kafirin-based bioactive film.^49^ The addition of OEO nanoemulsion increased the thickness of the films. This result can be attributed to the increase in the free volume of the film due to the addition of nanoemulsion (Fig. 2).

**Table 6:** Physical and mechanical properties of edible films

Type	Moisture content (%)	Thickness (mm)	Tensile strength TS (MPa)	Elongation at break, *E* (%)	Water vapor permeability (10^−5^ g Pa^−1^ m^−1^ s^−1^)
Control	12.12 ± 0.47	0.115 ± 0.01	19.72 ± 0.29	20.3 ± 0.14	9.44 ± 1.21
5%	10.44 ± 0.52	0.121 ± 0.02	16.38 ± 0.35	23.3 ± 0.22	7.24 ± 1.24
10%	8.63 ± 0.63	0.130 ± 0.02	14.85 ± 0.44	24.5 ± 0.24	6.18 ± 0.83
15%	7.12 ± 0.31	0.136 ± 0.01	13.12 ± 0.23	26.8 ± 0.18	5.34 ± 1.12

**Fig. 3:**
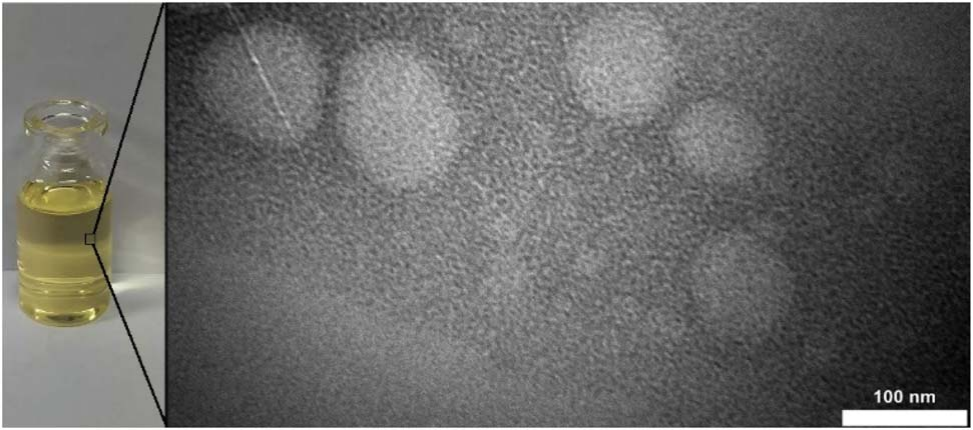
Transmission electron microscopy photographs of optimized formulation of OEO nanoemulsion.

The incorporation of OEO nanoemulsion increased the *E*% and decreased the TS and of films as compared with the control. So that the lowest amount of TS and the highest amount of *E*% correspond to the film containing 15% nanoemulsion. Reducing the interaction between polymer chains weakens intermolecular and intramolecular forces, which can lead to a decrease in the mechanical strength of the film.^50^ Similarly, a decrease in TS of film with increasing cinnamon essential oil content in sodium alginate/carboxymethyl cellulose films was observed by Han *et al.*^51^ The increase in the amount of *E*% film indicates that the flexibility and elasticity of the film has improved, which could be related to the plasticizing property of the EO. In research conducted by Erfanifar *et al.*, the mechanical properties of sage seed gum bioactive film were investigated after adding *Zataria multiflora* essential oil nanoemulsion. Their results revealed that with increasing concentration of EO, TS declined due to weakening film network cohesion.^52^ Azadbakht *et al.* also showed that increasing the amount of *Eucalyptus globulus* essential oil in chitosan film declines TS and increases *E*% (Fig. 3).^53^

There is a significant difference between the water vapor permeability of the films. Low water transmission rate could be a beneficial feature of edible films in food packaging industry to prevent losing or absorbing of moisture. Control film has a higher concentration of plasticizer such as glycerin and therefore has a higher WVR than films containing EO. Another reason could be the presence of OEO compounds, which due to their hydrophobic nature, can reduce water transfer. It has also been shown that the presence of lipid compounds in the film structure improves moisture resistance.^54^ A similar trend in the WVR values have been reported for chitosan film incorporated with *Zataria multiflora*^55^ and kafirin-based bioactive films containing citral and quercetin.^49^

## 4 Conclusion

This study demonstrated that RSM method is an effective tool for performing the optimization of formulations of OEO nanoemulsions by three different variables: SOR, sonication time and amplitude. The surfactant-to-oil ratio, ultrasonication time and power, all had a considerable effect on the droplet size of nanoemulsion. The results of DLS and TEM analysis confirmed the formation of nanoemulsion under optimal conditions. The antioxidant activity study showed that the OEO has good antioxidant activity compared to BHT. Besides, the edible film containing OEO has the potential for use as an edible film because of its suitable mechanical properties such as tensile capacity and tensile strength and also physical properties like water vapour permeability and solubility. Therefore, it can be concluded that edible films containing OEO may offer a feasible approach to enhancing industrial applications in food packaging and reducing spoilage.

## Author contributions

Azita Shafiei: conceptualization, data curation, investigation, writing; Javad Safaei-Ghomi: conceptualization, resource, writing and editing; Mehdi Mehran: software, formal analysis, validation and editing.

## Conflicts of interest

There are no conflicts to declare.

## Data Availability

The datasets produced and analyzed during this study are available from the corresponding author upon reasonable request.
